# Psychosocial Hazards in the Northern Territory Building and Construction Industry: A Profile of Job Demands and Job Resources in a Jurisdiction and Industry with High Rates of Suicide

**DOI:** 10.3390/ijerph21030334

**Published:** 2024-03-12

**Authors:** Nicholas Thompson, Adam Robertson, Rebecca Loudoun, Amanda Biggs, Keith Townsend

**Affiliations:** 1 MATES in Construction, Spring Hill, QLD 4004, Australia; nthompson@mates.org.au; 2 Centre for Work, Organisation and Wellbeing, Griffith University, Nathan, QLD 4111, Australia; r.loudoun@griffith.edu.au (R.L.); a.biggs@griffith.edu.au (A.B.); k.townsend@griffith.edu.au (K.T.)

**Keywords:** psychosocial hazards, work health and safety, suicide prevention, job control, supervisor task conflict, peer support, fly-in fly-out, drive-in drive-out, building, construction

## Abstract

The work environment for building workers in Australia’s Northern Territory (NT) is characterised by concerningly high rates of distress and suicide at both a jurisdictional and an industry level. Work-related psychosocial hazards are known antecedents of work-related distress and suicide, and more research is required to understand how these hazards impact workers in this unique building context. This paper examines the unique work environment in the NT building industry by comparing psychosocial hazards in the NT with those in the broader Australian building and construction industry. When comparing 330 NT self-report survey responses about psychosocial hazards in the workplace to 773 broader Australian building industry responses, supervisor task conflict for NT workers was more concerning, at 10.9% higher than the broader Australian cohort. Within the NT sample, comparisons between fly-in and fly-out/drive-in and drive-out (FIFO/DIDO) workers and non-FIFO/DIDO workers were also performed to determine specific local psychosocial hazards. When comparing FIFO/DIDO workers’ responses to their NT peers, role overload and supervisor task conflict were significantly higher, and co-worker and supervisor support were lower. In FIFO/DIDO environments, praise and recognition, procedural justice, and change consultation were at concerningly lower averages than the broader NT building and construction industry. These results suggest that the NT building and construction industry, and particularly FIFO/DIDO operations, require greater resourcing, investment, and focus on workplace mental health initiatives to improve the work environment and wellbeing of this workforce and mitigate hazards that can lead to distress and the high rates of occupational suicide found in this jurisdiction and industry.

## 1. Introduction

The impact of work-related psychosocial hazards on workers’ mental health is a serious work health and safety issue, requiring government, industry, and site-based investment. Psychosocial hazards in Australian workplaces are regulated through State and Territory jurisdictions with differing localised responses [1]. As a jurisdiction, the Northern Territory (NT) faces unique challenges and opportunities in formulating responses to the management and understanding of these work environment stressors. The NT’s small demographic size and decentralised population adds complexity to this management [1]. The NT and the Australian building and construction industry, more broadly, have jurisdictional and occupational rates of distress and suicide that are concerningly high [2]. This finding emphasises our need to better understand workers’ levels of exposure to different psychosocial hazards, which are known precursors of distress and suicide [3,4]. This article examines exposure to several psychosocial hazards self-reported by building and construction workers in the NT, in comparison with a broader cohort of building and construction workers throughout Australia, with the aim of identifying any unique factors in the industry that relate to the NT’s construction workforce and how it may require localised industry responses. This understanding will inform the development of targeted intervention strategies to improve mental health within the NT building environment.

**Figure 1:**
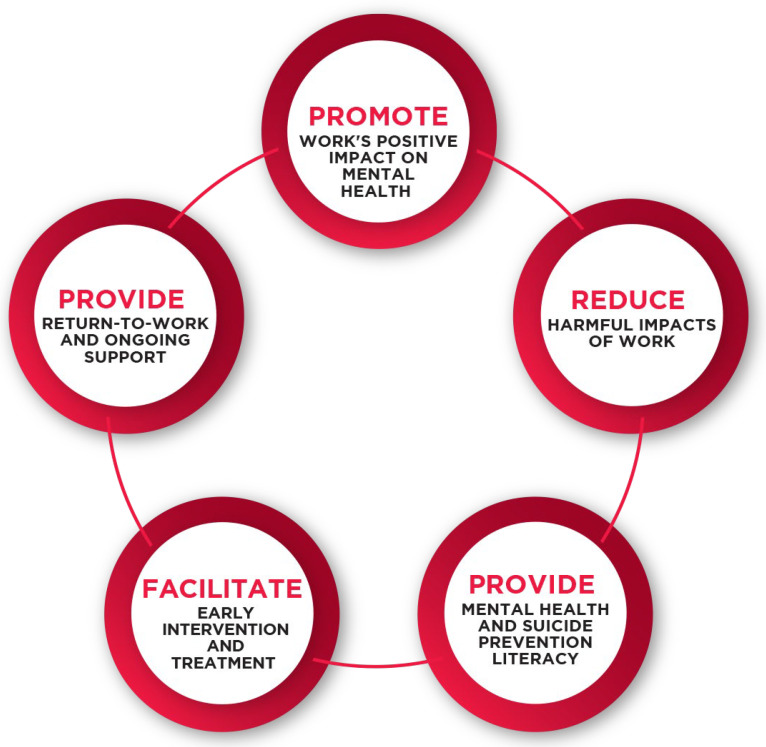
The five-pillar model for mental health interventions in the workplace in the building and construction industry (the Blueprint).

Using an existing industry-devised and endorsed framework, called the Blueprint for Better Mental Health and Suicide Prevention in the Building and Construction Industry (the Blueprint), the “Reduce Harmful Impacts of Work” domain within the Blueprint (refer to Figure 1) was investigated in this research [5,6]. The Blueprint framework, developed in 2016 and operationalised by MATES in Construction since 2019, is the overarching tool used by the industry to gauge the effectiveness and need for workplace mental health and suicide prevention initiatives [5,6]. The “Reduce Harmful Impacts of Work” domain aims to address work-related psychosocial hazards, which are known precursors of mental ill-health and suicide. Psychosocial hazards, also referred to as work-related stressors, are defined in line with job demands–resources (JD-R)theory [7] and occur when workers have insufficient job resources to meet their high job demands.

### 1.1. Suicide, Work-Related Stress, Distress, and Self-Harm in the Northern Territory

This research aims to better understand the levels of exposure to different psychosocial hazards within the NT building industry, which will inform the future development of targeted intervention strategies. This is a necessary step towards effectively addressing the concerning levels of distress and suicide within the NT, which is the highest of any jurisdiction in Australia [8]. Exposure to work-related psychosocial hazards is a contributing factor to suicide, acknowledged by the NT Government, which has specific plans and strategies for prevention to stem these unnecessary deaths [9]. The NT has several all-of-government approaches to suicide prevention, including dedicated funded services, such as MATES in Construction, to improve workplace mental health [10]. Localised responses from NT leaders and experts in suicide prevention and mental health also identify the role that workplaces have in the wellbeing of workers and the protective factors attached to work that can mitigate mental ill-health and suicide [11]. Critical to suicide prevention and workplace mental health initiatives in the NT is the use of local knowledge and experience in the design and formation of responses [12]. NT-specific trends in workplace mental health stressors largely relate to industry-specific workforces in the fields of health, social services, and government, where most NT residents are employed [13]. The building and construction workforce, however, does not have the same level of understanding of the collective industry’s work environment and associated mental health hazards. Where there is evidence, it is of a secondary nature; for example, focusing on the impacts of management styles with a view to increase productivity in local NT construction projects [14]. This evidence is strengthened by project-specific and trade-specific knowledge in managing the rostering in larger NT projects [15]. The substantial economic and human costs, including mental ill-health, of suicide in the building and construction industry from the perspective of cost and wellbeing is evidenced by Doran, Ling, and Milner [16]. Their study indicates a significant cost, separate from the collective community’s grief, of an incident of self-harm in the building and construction industry [16]. Doran, Ling, Milner, and Kinchin suggest that each fatality from self-harm and suicide results in a cost of AUD $2.72 million to the Northern Territory, with the government covering 97% of that cost [17]. The rates of suicide for male NT construction workers, from 2001–2019, have remained higher compared to suicide rates for males outside the building and construction industry [18]. Whilst the data available to make this analysis are modest and have multiple variables, they do imply an increase in the rates of suicide for construction workers in the NT over this period [18]. Although evidence demonstrates that high rates of suicide in the NT building and construction industry is a concern, the relationships between suicide and psychosocial hazards require further exploration.

### 1.2. Psychosocial Hazards in the Northern Territory Building and Construction Work Environment

According to JD-R theory, work-related psychosocial hazards are present when workers experience high job demands and low job resources [7]. Chronic exposure to high levels of job demands, such as work overload, long hours, role ambiguity, and conflict, exhausts workers’ physical and emotional energy, leading to burnout and mental ill-health. Job resources, such as job control and social support, are the aspects of work that enable workers to meet their job demands, achieve their goals, and promote personal and professional growth. Substantial research conducted in a variety of occupational groups has demonstrated that insufficient job resources in the presence of high job demands leads to mental ill-health, low morale, and poor performance [19]. Furthermore, the influential role of “upstream factors” such as leadership behaviours and organisational culture in shaping this connection has been well established (e.g., [20]).

Research conducted within the building and construction industry has also demonstrated support for the key tenets of JD-R theory (e.g., [21,22]). The NT building and construction industry, as part of the broader global construction industry, has evidenced and known workplace mental health stressors. High job demands, combined with low levels of job resources, such as poor supervisory relationships and peer support, produce high levels of stress that requires specific attention in the industry [5,6]. The extant literature applying JD-R theory has also established that low levels of job control is a primary and ongoing stressor for building and construction workers [23]. Similarly, role ambiguity and poor relationships are strong features of stress in the construction work environment [24]. Peer support and the perception of an understanding and trustworthy work environment is an additional known protective factor for workplace mental health [25]. This point is further evidenced in the experiences of workers themselves; having trusted and reliable peers and established relationships is known to strengthen construction workers’ sense of support and community when facing mental health adversity or distress [25]. Further to this sense of support, there are fewer negative perceptions of reprimand, blame, or vulnerability in needing support or assistance from trusted peers when disclosing workplace mental health challenges [26]. Specific construction projects in the NT that have long been completed and had a problematic workplace mental health record add to this body of evidence [15]. Factors associated with these problematic ways of structuring the work environment, whilst historical, demonstrate the need to factor in the transient nature of many construction workers in the NT and the impact of fly-in and fly-out or drive-in and drive-out work (FIFO/DIDO) [27]. While more research is needed, there is some evidence to suggest that working under FIFO/DIDO and subcontractor working arrangements is linked with a higher exposure to psychosocial hazards and poorer mental health. Research has demonstrated that FIFO/DIDO workers report high levels of psychological distress, suicide intent, and rates of death-by-suicide [28,29,30].

As with the rise in FIFO/DIDO operations, the utilisation of subcontractor relationships is becoming increasingly important within the building and construction industry. Limited research understanding the impact of subcontractor relationships on workers’ mental health has been conducted; nonetheless, it does suggest that subcontractors experience a heightened risk of physical and psychosocial hazards at work due to the precarious nature of their employment. For instance, Valluru and colleagues [31] reviewed research demonstrating that subcontractor employees are at higher risk of injury, are subjected to different work pressures, are less protected by institutional safety mechanisms and regulatory actions, and undertake riskier work. Their qualitative study specified three categories of factors that were discussed as reasons why subcontractor employees experience safety differently to other employees: fixed conditions (e.g., project, workforce, industry regulations, collective organisation); on-site conditions (e.g., leadership, trust, isolated teams, goals/expectations, and commercial arrangements); and outcomes (e.g., project risk, site safety, and mental wellbeing). Of most interest to the current research were the findings that included a higher presence of psychosocial hazards, such as poor leadership, and the compounding issue of precarious employment within an occupation that is already at higher risk of mental ill-health and suicide [31].

This paper aims to improve our knowledge of the levels of psychosocial hazards experienced by workers within the NT building and construction industry, compared to the Australian building and construction industry more broadly. This understanding is a necessary step toward developing localised, targeted intervention responses. Addressing work-related psychosocial hazards is important, given their impact on a range of adverse outcomes, including poor productivity, mental health, and suicide. As demonstrated, these are at alarmingly high levels within the NT building and construction industry, requiring localised and targeted responses. On this basis, we propose the following hypotheses:

**Hypothesis** **1:** *NT participants will report significantly higher job demands and significantly lower job resources than workers in the broader Australian building and construction industry*.

Amongst the NT participants, it is also expected that those working within FIFO/DIDO settings will also be exposed to greater psychosocial hazards, given the unique FIFO/DIDO working environment.

**Hypothesis** **2:** *NT participants working within FIFO/DIDO settings will report significantly higher job demands and significantly lower job resources than non-FIFO/DIDO workers*.

Finally, amongst the NT participants, it is also expected that subcontractors will be exposed to greater psychosocial hazards.

**Hypothesis** **3:** *NT participants working as subcontractors will report significantly higher job demands and significantly lower job resources than non-subcontractors*.

## 2. Materials and Methods

### 2.1. Sample

Data were collected from building and construction workers across 21 construction sites within all Australian States and Territories from August 2022 to April 2023 (MATES in Construction 2022–2023 Data Collection Period). To increase the generalisability of the results, participants were recruited from various principal contractors and subcontractors across the industry, including commercial, residential, civil construction, and interior fit-out organisations. These organisations represent many of Australia’s most common construction works [32]. General invitations to participate in the research were made by MATES in Construction field officers during toolbox talks and industry awareness information sessions held across the sites either before the commencement of work or at an assigned time prescribed by the principal contractor of that site. Participation was voluntary, with information sheets explaining the non-compulsory nature and confidentiality attached to the survey. Surveys were distributed in paper format with the added benefit of collective peer engagement encouraging anonymity and the need for return.

A total of 1103 completed surveys were returned, including 330 respondents from the NT and 773 from all the remaining Australian States and Territories. Due to the dynamic nature of project work on a construction site, it is not possible to determine an exact response rate. However, daily site reports for each of the 21 construction sites approximate an overall response rate of around 85%. It was as low as 50% and as high as 100% in some workplace settings. Individual workgroups had an allocated site contact, normally a workplace health and safety representative who, after the completion of the surveys, received an aggregated report with recommendations for an improvement plan if areas of concern were identified, to assist with the Blueprint alignment of the site or company undertaking the survey.

### 2.2. People at Work—Construction Survey (PAW-Con)

Data on psychosocial hazards were gathered using the 36-item People at Work—Construction Survey (PAW-Con). The PAW-Con is an empirically validated measure of the work environment underpinned by JD-R theory [7,33], and it has been used previously in research investigating psychosocial hazards within the building and construction industry [34]. The PAW-Con survey builds upon existing research from Safe Work Australia and all State and Territory governments throughout Australia in their development of the People at Work (PAW) survey [35]. The original PAW survey was modified in a previous two-part study to create a new measure that used appropriate and congruent language attached to the building and construction industry and evaluated aspects of work that were unique to the psychosocial factors found in the construction work environment [33].

The PAW-Con asks participants to report on 11 job demands and job resources relevant to the construction industry: role overload (4 items; α = 0.87; e.g., “*I am pressured to work long hours*”), role ambiguity (4 items; α = 0.87; e.g., “*I am clear what is expected of me on this job*”), role conflict (3 items; α = 0.88; e.g., “*Different people on this job expect conflicting things from me*”), job control (3 items; α = 0.82; e.g., “*I have a choice in deciding what I do on this job*”), co-worker support (3 items; α = 0.91; e.g., “*If the work gets difficult, my co-workers will help me*”), supervisor support (3 items; α = 0.95; e.g., “*If the work gets difficult, my direct supervisor on this job will help me*”), supervisor task conflict (3 items; α = 0.92; e.g., “*I have conflict with my direct supervisor on this site about the work I do*”), supervisor relationship conflict (3 items; α = 0.94; e.g., “*There are bad feelings between me and my direct supervisor on this site*”), praise and recognition (3 items; α = 0.96; e.g., “*My direct supervisor on this job gives me sufficient credit for my hard work*”), procedural justice (3 items; α = 0.87; e.g., “*Supervisors consistently follow the policies and procedures set out for this site*”), and change consultation (4 items; α = 0.89; e.g., “*I am consulted about proposed changes on this job*”). All job resource items were scored on a 7-point scale, ranging from 1 (*Never*) to 7 (*Always*), with higher scores indicating more resources. Job demand items were also scored on a 7-point scale, ranging from 1 (*Never*) to 7 (*Always*); higher scores indicated greater job demands, except for role ambiguity, where items were subsequently reverse-coded, such that higher scores indicated greater role ambiguity.

### 2.3. Statistical Analyses

To establish workplace mental health trends in the NT and compare them against the broader Australian building and construction work environment, the data were analysed using a one-way between-groups Multivariate Analysis of Variance (MANOVA), aiming to identify significant differences in job demands and job resources among the groups of interest, such as FIFO/DIDO versus non-FIFO/DIDO employees within the NT, subcontractor versus non-subcontractor employees within the NT, and comparisons between NT and non-NT employees. Table 1 outlines the mean, standard deviation, reliability, and intercorrelations across all job demands and job resources.

## 3. Results

### 3.1. Demographics of Participants

The overall sample of 1103 participants was separated into two distinct samples to address this study’s aims. The NT cohort consisted of 330 workers employed in the building and construction industry. Most respondents were male (87%), born in Australia (76%), had ten or more years of industry experience (57% of employees who reported) and were an average age of 37 years old. The sample was primarily individuals employed by the principal contractor (59%) or subcontractors (29%). Employee role descriptions included a range of positions, such as tradesperson (21%), apprentice (21%), and operator (11%). The direct supervisor of the respondents was predominately the site manager (35%), followed by the leading hand (25%) or another manager such as a construction or project manager (18%). Finally, the sample included 148 employees (45%) from FIFO/DIDO sites.

The broader Australian sample consisted of 773 workers employed in the building and construction industry throughout all Australian States and Territories (excluding the NT). Consistent with the NT-based sample, this larger group was also predominantly male (88%), born in Australia (68%), had ten or more years of industry experience (67% of employees who reported) and were an average age of 42 years old. They were also employed in a range of positions, such as tradesperson (30%) and operator (22%), while fewer were apprentices (1.8%). In contrast to the NT-based sample, the direct supervisor of this group was predominately the leading hand (27%), and most respondents classified themselves as subcontractors (49%). The sample also included a larger proportion of FIFO/DIDO employees (76%).

### 3.2. Job Demands within the NT

Upon evaluating the 17 items that gauge the five employee job demands, it is evident that NT employees (N = 330) largely feel they are navigating through a manageable work environment. Specifically, they reported relatively low levels of role overload, with an average score of 2.62 out of 7; role ambiguity at 1.78, role conflict at 2.55, friction with supervisors about how tasks are completed at 2.37, and relational conflicts with supervisors at 1.63 (the classification of job demands and job resources as low (1.00–3.00), moderate (3.01–4.99), or high (5.00–7.00) is in line with industry practice and the People at Work (PAW) comprehensive reporting template (https://www.peopleatwork.gov.au/assets/pdf/People%20at%20Work%20-%20Comprehensive%20report%20example%20-%20excerpt.pdf (accessed on 14 December 2023))). 

### 3.3. Job Resources within the NT

When looking at how well resourced NT workers felt they were to perform their role, a reasonably positive picture emerges. Most workers felt they had a moderate level of control over their jobs, with an average score of 4.60 out of 7. There was also a strong sense of camaraderie and acknowledgment, as shown by higher scores in areas like co-worker support (5.70 out of 7), supervisor support (5.72 out of 7), praise and recognition (5.27 out of 7), procedural justice (5.85 out of 7), and change consultation (5.30 out of 7).

### 3.4. Differences between FIFO/DIDO and Non-FIFO/DIDO Employees within the NT

Within the sample, 148 workers indicated that they worked at FIFO/DIDO sites within the NT. Compared with their non-FIFO/DIDO counterparts (N = 149), these workers reported significant differences in their job demands and resources; *F*(11, 264) = 3.33, *p* ≤ 0.001, Wilks’ Lambda = 0.88, partial eta squared = 0.12. When reviewing the results for job demands separately, FIFO/DIDO workers in the NT reported significantly higher role overload (17.9%; M_Diff_ = 0.43, *p* = 0.01, CI_95%_ = [0.13, 0.73]) and supervisor task conflict (26.0%; M_Diff_ = 0.56, *p* ≤ 0.00, CI_95%_ = [0.24, 0.88]). However, responses for role ambiguity, role conflict, and supervisor relationship conflict were not significantly different between the two NT samples.

FIFO/DIDO workers also reported significantly lower levels of important job resources, including reduced co-worker support (−6.0%; M_Diff_ = −0.35, *p* ≤ 0.02, CI_95%_ = [−0.66, −0.05]), supervisor support (−7.1%; M_Diff_ = −0.42, *p* ≤ 0.01, CI_95%_ = [−0.73, −0.11]), praise and recognition (−8.4%; M_Diff_ = −0.46, *p* ≤ 0.01, CI_95%_ = [−0.81, −0.11]), procedural justice (−8.5%; M_Diff_ = −0.52, *p* ≤ 0.00, CI_95%_ = [−0.76, −0.27]), and change consultation (−13.2%; M_Diff_ = −0.74, *p* ≤ 0.00, CI_95%_ = [−1.06., −0.43]), compared to their non-FIFO/DIDO counterparts. The reported levels of job control were not significantly different between the two NT samples. Table 2 outlines the results for FIFO/DIDO and non-FIFO/DIDO employees within the NT.

### 3.5. Differences between Subcontractor and Non-Subcontractor Employees within the NT

Within the sample, 97 participants identified that they worked for a subcontractor within the NT. Parsing their feedback, these workers reported significant differences in their reported job demands and resources compared to their non-subcontractor peers; (N = 218), *F*(11, 283) = 1.81, *p* = 0.05, Wilks’ Lambda = 0.93, partial eta squared = 0.07. Comparing results for job demands and job resources separately, subcontractors reported significantly reduced job control (−7.8%; M_Diff_ = −0.37, *p* = 0.05, CI_95%_ = [−0.74, −0.01]) compared to their non-subcontractor peers. All other job demands and resources were not significantly different between the two NT samples. Table 3 outlines the results for subcontractor and non-subcontractor employees within the NT.

### 3.6. Differences between NT and Non-NT Employees

A comparison between NT employees (N = 330) and employees from the remaining Australian States and Territories (N = 773) suggests minor differences in reported job demands and resources; *F*(11, 1020) = 2.95, *p* ≤ 0.001, Wilks’ Lambda = 0.97, partial eta squared = 0.03. Comparing their results for job demands and resources separately, NT employees reported significantly greater perceived supervisor task conflict (11.7%; M_Diff_ = 0.25, *p* ≤ 0.00, CI_95%_ = [0.09, 0.41]) compared to their non-NT counterparts. All other job demands and resources were not significantly different between the two samples. Table 4 outlines the results for NT and non-NT employees.

## 4. Discussion

Overall, our findings provided strong support for our hypothesis that FIFO/DIDO workers in the NT would report more psychosocial hazards than non-FIFO/DIDO workers. The results indicate that FIFO/DIDO workers in the NT experienced more role overload and supervisor task conflict than their non-FIFO/DIDO peers, as well as reduced co-worker and supervisor support, praise and recognition, procedural justice, and change consultation. However, there was limited support for our hypotheses that NT workers and NT subcontractors would report greater job demands and fewer job resources than non-NT workers and non-NT subcontractors. The results suggest that supervisor task conflict was higher for NT workers than the broader Australian cohort, and subcontractors in the NT reported less job control than their non-subcontractor peers. However, all other job demands and resources remained statistically similar between these groups. Compared with the broader Australian building and construction industry, the differential impact of psychosocial hazards on NT workers, particularly FIFO/DIDO workers, has been hitherto neglected in past research addressing the work environment but is likely to have important policy and practice implications for regulators, organisations operating in the NT, and employee representatives.

### 4.1. The Supervisory Relationship in the Northern Territory Construction Work Environment

The findings of this study on the Northern Territory (NT) building and construction industry trends in workplace mental health demonstrate a significant improvement from studies undertaken throughout the duration of COVID-19 [36]. The NT sample itself is small in scale both for the NT and the broader industry, implying that more intimate teams with established relationships can appear to influence workers’ perceptions of their job resources, job demands, and mental health stressors [37]. The ratio of workers employed by a principal contractor versus subcontractors similarly influences this relationship. Given the lower percentage of subcontractor employees within the NT sample, the outcome of subcontractor employees having less job control adds to the complexity of their work environment and supervisory relationships [36].

Small workgroups with closer relationships, hypothetically, can add to the need for individual workers to perform multiple job tasks and have their supervisor task expectations influenced by the scale of the worksite and workgroup [37]. Further to the smaller scale of these workgroups, the industrial dynamics of workgroups are considerably different across the NT. Enterprise bargaining agreements in the NT are rare compared to the broader Australian industry, changing the dynamics and environment for employees and subcontractors [38,39]. The NT sample has a lower proportion of subcontractors, and the identified supervisor of a workgroup was more likely to be a site manager rather than a leading hand, which is the case in the Australian sample. The managerial role of a site supervisor compared to a leading hand, who is more likely to have trade-based expertise and knowledge, has the potential to directly impact supervisor task conflict in the NT construction work environment [40].

This study demonstrates the need for clear policies and processes for managing psychosocial hazards in the NT building and construction work environment. Supervisor task conflict refers to conflict over how work is performed onsite. It contrasts with supervisor relationship conflict, which refers to bad feelings with a personal, rather than a problem, focus. In high-risk and trades-based roles, control over how work is performed can be perceived by supervisors as an untenable goal; however, industry representatives need to think more broadly about what this means at the day-to-day site level [41]. Tradespeople have expertise in their industry and embodying choice by exercising this expertise could dramatically shift the rates of distress, which is achievable through self-efficacy and systematic changes to job flow [42]. The existence of smaller, more established teams and the geographical and social context of the NT construction environment means that workers are required to do many different jobs that would normally have specific clarity in the broader Australian context. Industrial instruments that provide specificity to trades-based roles, conditions and expectations, and minimum standards in relation to workplace health and safety are more absent in the NT. This argument is hard to quantify with such a small sample, however, supervisor task conflict is known to have considerable negative impacts in the construction work environment, including impacts on physical and mental safety. The NT data that demonstrated this relationship contrasted with results for the broader Australian sites [43]. With supervisor task conflict representing such a variance from the Australian cohort, and at an even more alarming rate in FIFO/DIDO work environments in the NT, it is critical that sites, companies, the industry, and government work together to develop industry-based training for the tradespeople managing diverse teams in diverse work contexts that incorporate the nuances of subcontracting and the trades-based work environment of the building and construction industry in the NT.

### 4.2. Peer Support in the Northern Territory Building and Construction Industry

Peer support has been evidenced to be critical to psychosocial safety in the construction work environment, and this sample demonstrated minimal concern with this item compared to previous COVID-19 data [25,36]. This sample in the NT was garnered from sites that had a strong peer support network and a training program attached to suicide prevention and mental health awareness, including accreditation with MATES in Construction [25]. Further exploration of work environments where these factors are not present could demonstrate how significant peer support is to worker wellbeing and give broader clarity to peer support in the Northern Territory building and construction industry. The impact of a strong peer support network in FIFO/DIDO environments also warrants additional research to determine whether these factors can influence workers’ perceptions of job control, change consultation, and procedural justice. 

Furthermore, peer support can take many forms in challenging work environments [44]. As one example, formal peer support networks have provided paramedics with a great deal of assistance in mitigating psychosocial hazards [45]. This can be achieved through a range of different mechanisms, for example, informal peer-to-peer support for employees who are having difficulty coping [46]; a slightly more structured system of peer support, for example, the mates supporting mates system advocated by MATES in Construction; or formalised systems that provide direct feedback to senior management and human resource (HR) departments. This formal system, in theory, should provide feedback in a timely manner about issues that are arising, allowing the HR personnel to intervene and mitigate the hazards [47].

### 4.3. Regional and Remote Work Environments in the Northern Territory Building and Construction Industry

FIFO/DIDO work environments accounted for nearly half of all participants in the Northern Territory sample. The work environment was of particular concern for this cohort, with role overload, supervisor task conflict, procedural justice, and change consultation being significantly higher than their non-FIFO/DIDO peers. The challenge of FIFO/DIDO work environments and their associated psychosocial hazards are not unique to the NT work environment [5,6]. Workplace health and safety committees have a clear responsibility for managing and mitigating these unique factors that can lead to distress, mental health challenges, and suicide [48]. Coupled with broader work factors such as their isolation from family-based supports and the lack of opportunities to recover from work, the FIFO/DIDO work environment presents significant hazards for workers that require additional exploration and an industry-wide investment in mitigation [15]. The lower averages of co-worker support in FIFO/DIDO work environments corroborate previous studies that found isolation to be a major contributor to distress in FIFO/DIDO work environments [48].

## 5. Limitations and Future Research

Our sample was constrained in several ways. The NT cohort, comparatively, was small in scale both in relation to the local industry and the broader Australian building and construction industry. Future research would benefit from additional data collection and a larger sample size; this would allow for a deeper exploration of the variances in supervisor task conflict represented in the NT data due, for example, to the size and nature of the construction work, such as civil, commercial, and maintenance work. Future research focussing on the influence of broader workplaces characteristics on psychosocial hazards would directly benefit the planning and focus of the NT government and key stakeholders in the NT’s building and construction industry in terms of hazard mitigation and suicide prevention.

There is also a need for future research to explore the use of on-site programs that develop and maintain a formal peer support network [25]. Contrasting our results with the responses of sites that do not have formal peer support networks in place has the potential to see if peer support is in fact as commonplace and evidenced as reported in the NT sample and whether it requires particular attention in FIFO/DIDO work environments.

An additional limitation of our sample is the use of survey data only. Although we can adequately measure many factors that contribute to job demands and job resources in this context, combining this survey data with qualitative data would provide a greater understanding of the psychosocial hazards identified in this unique working environment and expand their meaning. For example, there would be great value in performing qualitative research to better understand the range of issues that employees and their supervisors face as time progresses on projects. A qualitative study that draws on the broader experiences of NT workers could also provide further insight into differences found for FIFO/DIDO workers, such as how the quality of relationships is influenced by shifts that require longer periods both at, and away from, the workplace. This is just one of the context-specific research areas that would benefit substantially from a structured programme of qualitative studies.

Finally, it is also important to acknowledge that the elements of the mental health framework outlined in Figure 1 are interconnected. The underlying purpose of the framework was to work towards a broader systems-based approach to workplace mental health in the building and construction work environment [34]. The focus of this research was the "Reduce Harmful Impacts of Work” element of the Blueprint framework and, specifically, to identify the psychosocial hazards for building and construction workers in the NT to highlight unique factors that may require localised industry responses. Future research is needed to fully understand how these psychosocial hazards interact with the other four elements of the framework.

## 6. Conclusions

Workplace stressors in the NT building and construction industry largely replicate the trends in the broader Australian building and construction industry, and mainly the impact of job control on the work environment. Supervisor task conflict in the NT represents a concerning difference from that of the broader Australian building and construction industry and it is even more problematic in FIFO/DIDO workgroups. Supervisor task conflict requires localised NT-specific initiatives and responses to mitigate this hazard and support and enhance individual tradespeople and NT companies in leadership development, management skills, and systems-based policies. 

The literature and the data within this study suggest that FIFO/DIDO work environments have significant mental health stressors that require broader exploration in future NT worker samples and in the industry and government responses to FIFO/DIDO projects. The evidence of improved supervisory relationships with respect to previous studies throughout COVID-19 requires qualitative data to better understand why this shift has taken place and what strengths exist in the NT that can be replicated in job sites to improve mental health and wellbeing, particularly in smaller work environments, such as the residential construction sector. There is also a need to explore why supervisor relationships have been enhanced while supervisor task conflict is of concern. Implementing better hazard controls regarding job control is a clear priority for stakeholders in the NT building and construction industry to mitigate and prevent distress and self-harm, as evidenced through these data. Investigation of peer support networks as a protective factor for psychosocial safety is also critical and requires the inclusion of contrasting sites with no peer support network present, to confidently determine the impact of these networks on improved mental health and wellbeing. The NT building and construction work environment has a unique psychosocial profile of strong peer and supervisor support. However, it also has problematic supervisor task conflicts and challenging FIFO/DIDO work environments, with concerning psychosocial outcomes for these workers compared to their peers. 

## Figures and Tables

**Table 1:** Intercorrelations among job demands and job resources.

	1	2	3	4	5	6	7	8	9	10	11	M	SD
Role Overload	(0.87)											2.69	1.28
2. Role Ambiguity	0.22 **	(0.87)										1.75	0.84
3. Role Conflict	0.54 **	0.23 **	(0.88)									2.54	1.34
4. Job Control	−0.16 **	−0.34 **	−0.12 **	(0.82)								4.57	1.48
5. Co-Worker Support	−0.29 **	−0.28 **	−0.32 **	0.34 **	(0.91)							5.73	1.27
6. Supervisor Support	−0.33 **	−0.33 **	−0.35 **	0.38 **	0.55 **	(0.95)						5.70	1.41
7. Supervisor Task Conflict	0.33 **	0.20 **	0.44 **	−0.07 *	−0.24 **	−0.36 **	(0.92)					2.21	1.22
8. Supervisor Relationship Conflict	0.25 **	0.21 **	0.39 **	−0.13 **	−0.24 **	−0.42 **	0.59 **	(0.94)				1.57	0.99
9. Praise and Recognition	−0.28 **	−0.34 **	−0.27 **	0.40 **	0.45 **	0.67 **	−0.35 **	−0.36 **	(0.96)			5.22	1.58
10. Procedural Justice	−0.34 **	−0.42 **	−0.38 **	0.39 **	0.47 **	0.69 **	−0.45 **	−0.47 **	0.67 **	(0.87)		5.78	1.17
11. Change Consultation	−0.35 **	−0.44 **	−0.37 **	0.42 **	0.49 **	0.55 **	−0.33 **	−0.28 **	0.58 **	0.66 **	(0.89)	5.17	1.41

Note: N = 1103; reliability coefficients (α) are in parentheses along the diagonal; * *p* < 0.05, ** *p* < 0.01.

**Table 2:** Differences between FIFO/DIDO and non-FIFO/DIDO employees within the NT.

Job Demand/ Resource	Sample	Mean (SD)	Mean Difference	*F* Value	*P* [CI_95%_]
Role Overload	FIFO/DIDO vs. Non-FIFO/DIDO	2.84 (1.43) 2.41 (1.06)	0.43	8.13 **	0.00 [0.13, 0.73]
Role Ambiguity	FIFO/DIDO vs. Non-FIFO/DIDO	1.85 (.83) 1.72 (.75)	0.13	1.83	0.18 [−0.06, 0.32]
Role Conflict	FIFO/DIDO vs. Non-FIFO/DIDO	2.59 (1.25) 2.47 (1.14)	0.12	0.65	0.42 [−0.17, 0.40]
Job Control	FIFO/DIDO vs. Non-FIFO/DIDO	4.60 (1.50) 4.67 (1.46)	−0.07	0.14	0.71 [−0.42, 0.28]
Co-Worker Support	FIFO/DIDO vs. Non-FIFO/DIDO	5.51 (1.44) 5.86 (1.09)	−0.35	5.27 *	0.02 [−0.66, −0.05]
Supervisor Support	FIFO/DIDO vs. Non-FIFO/DIDO	5.50 (1.50) 5.92 (1.10)	−0.42	7.00 *	0.01 [−0.73, −0.11]
Supervisor Task Conflict	FIFO/DIDO vs. Non-FIFO/DIDO	2.71 (1.45) 2.15 (1.21)	0.56	12.10 **	0.00 [0.24, 0.88]
Supervisor Relationship Conflict	FIFO/DIDO vs. Non-FIFO/DIDO	1.75 (1.23) 1.60 (1.12)	0.15	1.12	0.29 [−0.13, 0.43]
Praise and Recognition	FIFO/DIDO vs. Non-FIFO/DIDO	5.02 (1.58) 5.48 (1.37)	−0.46	6.63 *	0.01 [−0.81, −0.11]
Procedural Justice	FIFO/DIDO vs. Non-FIFO/DIDO	5.58 (1.16) 6.09 (.90)	−0.52	16.99 **	0.00 [−0.76, −0.27]
Change Consultation	FIFO/DIDO vs. Non-FIFO/DIDO	4.91 (1.54) 5.65 (1.08)	−0.74	21.54 **	0.00 [−1.06, −0.43]

Note. N = 139 (FIFO/DIDO); *N* = 137 (non-FIFO/DIDO); * *p* < 0.05, ** *p* < 0.01.

**Table 3:** Differences between subcontractor and non-subcontractor employees within the NT.

Job Demand/ Resource	Sample	Mean (SD)	Mean Difference	*F* Value	*P* [CI_95%_]
Role Overload	Subcontractor vs. Non-Subcontractor	2.68 (1.23) 2.60 (1.29)	0.08	0.28	0.60 [−0.23, 0.40]
Role Ambiguity	Subcontractor vs. Non-Subcontractor	1.76 (0.75) 1.83 (0.90)	−0.08	0.50	0.48 [−0.28, 0.13]
Role Conflict	Subcontractor vs. Non-Subcontractor	2.52 (1.26) 2.51 (1.19)	0.02	0.01	0.91 [−0.28, 0.32]
Job Control	Subcontractor vs. Non-Subcontractor	4.38 (1.54) 4.75 (1.46)	−0.37	4.05 *	0.05 [−0.74, −0.01]
Co-Worker Support	Subcontractor vs. Non-Subcontractor	5.68 (1.37) 5.71 (1.26)	−0.02	0.02	0.90 [−0.34, 0.30]
Supervisor Support	Subcontractor vs. Non-Subcontractor	5.78 (1.39) 5.70 (1.33)	0.08	0.22	0.64 [−0.25, 0.41]
Supervisor Task Conflict	Subcontractor vs. Non-Subcontractor	2.36 (1.55) 2.43 (1.25)	−0.07	0.15	0.70 [−0.40, 0.27]
Supervisor Relationship Conflict	Subcontractor vs. Non-Subcontractor	1.60 (1.14) 1.70 (1.16)	−0.10	0.48	0.49 [−0.38, 0.18]
Praise and Recognition	Subcontractor vs. Non-Subcontractor	5.38 (1.57) 5.18 (1.51)	0.20	1.11	0.29 [−0.17, 0.58]
Procedural Justice	Subcontractor vs. Non-Subcontractor	5.86 (1.14) 5.83 (1.05)	0.03	0.05	0.83 [−0.24, 0.29]
Change Consultation	Subcontractor vs. Non-Subcontractor	5.07 (1.39) 5.39 (1.39)	−0.32	3.41	0.07 [−0.66, 0.02]

Note. N = 95 (subcontractor); *N* = 200 (non-subcontractor); * *p* < 0.05.

**Table 4:** Differences between NT and non-NT employees.

Job Demand/ Resource	Sample	Mean (SD)	Mean Difference	*F* Value	*P* [CI_95%_]
Role Overload	NT vs. Non-NT Employees	2.59 (1.27) 2.71 (1.27)	−0.11	1.77	0.18 [−0.28, 0.05]
Role Ambiguity	NT vs. Non-NT Employees	1.80 (0.84) 1.72 (0.82)	0.08	1.94	0.16 [−0.03, 0.19]
Role Conflict	NT vs. Non-NT Employees	2.49 (1.21) 2.52 (1.35)	−0.04	0.16	0.69 [−0.21, 0.14]
Job Control	NT vs. Non-NT Employees	4.65 (1.50) 4.56 (1.46)	0.09	0.79	0.37 [−0.11, 0.29]
Co-Worker Support	NT vs. Non-NT Employees	5.72 (1.28) 5.76 (1.25)	−0.04	0.24	0.63 [−0.21, 0.13]
Supervisor Support	NT vs. Non-NT Employees	5.74 (1.33) 5.71 (1.42)	0.03	0.10	0.75 [−0.16, 0.22]
Supervisor Task Conflict	NT vs. Non-NT Employees	2.39 (1.34) 2.14 (1.16)	0.25	9.07 **	0.00 [0.09, 0.41]
Supervisor Relationship Conflict	NT vs. Non-NT Employees	1.64 (1.14) 1.53 (0.91)	0.11	2.74	0.10 [−0.02, 0.24]
Praise and Recognition	NT vs. Non-NT Employees	5.27 (1.51) 5.19 (1.61)	0.08	0.55	0.46 [−0.13, 0.29]
Procedural Justice	NT vs. Non-NT Employees	5.86 (1.07) 5.77 (1.19)	0.08	1.12	0.29 [−0.07, 0.24]
Change Consultation	NT vs. Non-NT Employees	5.31 (1.39) 5.13 (1.40)	0.18	3.63	0.06 [−0.01, 0.37]

Note. N = 307 (NT employees); *N* = 725 (non-NT employees); ** *p* < 0.01.

## Data Availability

Some or all data, models, or code that support the findings of this study are available from the corresponding author upon reasonable request and within constraints of ethical clearance.
